# Remarkably low host specificity in the bat fly *Penicillidia fulvida* (Diptera: Nycteribiidae) as assessed by mitochondrial *COI* and nuclear *28S* sequence data

**DOI:** 10.1186/s13071-022-05516-z

**Published:** 2022-10-27

**Authors:** Taylor B. Verrett, Paul W. Webala, Bruce D. Patterson, Carl W. Dick

**Affiliations:** 1 Department of Biology, Western Kentucky University, Bowling Green, KY 42101 USA; 2 Department of Forestry and Wildlife Management, Maasai Mara University, Narok, 20500 Kenya; 3 Negaunee Integrative Research Center, Field Museum of Natural History, Chicago, IL 60605 USA

**Keywords:** Bat flies, Chiroptera, Ectoparasites, Kenya, Nycteribiidae, Host specificity, Host-parasite interactions, Cryptic species, Molecular biology

## Abstract

**Background:**

The recognition and delineation of morphologically indistinguishable cryptic species can have broad implications for wildlife conservation, disease ecology and accurate estimates of biodiversity. Parasites are intriguing in the study of cryptic speciation because unique evolutionary pressures and diversifying factors are generated by ecological characteristics of host-parasite relationships, including host specificity. Bat flies (Diptera: Nycteribiidae and Streblidae) are obligate, hematophagous ectoparasites of bats that generally exhibit high host specificity. One rare exception is *Penicillidia fulvida* (Diptera: Nycteribiidae), an African bat fly found in association with many phylogenetically distant hosts. One explanation for *P. fulvida*’s extreme polyxeny is that it may represent a complex of host-specific yet cryptic species, an increasingly common finding in molecular genetic studies of supposed generalist parasites.

**Methods:**

A total of 65 *P. fulvida* specimens were collected at 14 localities across Kenya, from bat species representing six bat families. Mitochondrial cytochrome* c* oxidase subunit 1 (*COI*) and nuclear *28S* ribosomal RNA (rRNA) sequences were obtained from 59 specimens and used to construct Bayesian and maximum likelihood phylogenies. Analysis of molecular variance was used to determine how genetic variation in *P. fulvida* was allocated among host taxa.

**Results:**

The *28S* rRNA sequences studied were invariant within *P. fulvida*. Some genetic structure was present in the *COI* sequence data, but this could be more parsimoniously explained by geography than host family.

**Conclusions:**

Our results support the status of *P. fulvida* as a rare example of a single bat fly species with primary host associations spanning multiple bat families. Gene flow among *P. fulvida* utilizing different host species may be promoted by polyspecific roosting behavior in bats, and host preference may also be malleable based on bat assemblages occupying shared roosts. The proclivity of generalist parasites to switch hosts makes them more likely to vector or opportunistically transmit pathogens across host species boundaries. Consequently, the presence of polyxenous bat flies is an important consideration to disease ecology as bat flies become increasingly known to be associated with bat pathogens.

**Graphical Abstract:**

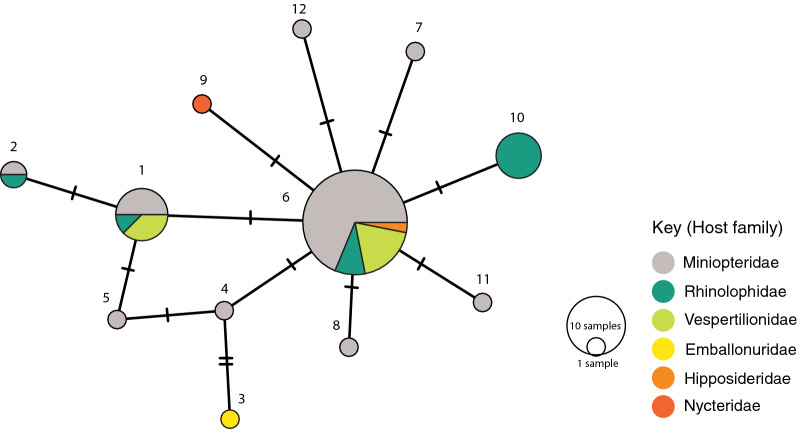

## Introduction

Although the species is a basic unit of organization in biology and is of fundamental importance in the study of ecology and evolution, the definition of a species is famously contentious. At least 32 species concepts have been described [1], but these are often variations on a few themes [2]: (i) the ability to successfully reproduce (e.g. the Biological Species Concept [3]); (ii) distinctive morphology (e.g. the Morphological Species Concept [4]); and (iii) shared evolutionary descent (e.g. the Phylogenetic Species Concept [5]). An integrative approach to species delimitation addressing several species concepts is often desirable, but some criteria are difficult to satisfy in practice or categorically inapplicable to certain study systems [2].

The limited reach of species concepts is demonstrated by cryptic species that are morphologically indistinguishable but genetically and often ecologically distinct [6, 7]. The evolutionary processes associated with cryptic speciation are variable, with cryptic speciation known to occur in allopatry [8], sympatry [9] and parapatry [10]. Recent advances in molecular systematics have significantly increased the rate of discovery of cryptic species [6, 7]. The recognition and delimitation of cryptic species is not only critical to accurately quantifying biodiversity, but misidentification of cryptic species complexes as single species can have profound consequences for wildlife conservation and disease ecology [6, 7].

Parasites are compelling candidates for the study of cryptic speciation because they experience evolutionary pressure from their hosts, which may be strong enough to result in speciation [11–13]. The integration of molecular techniques in delimiting parasite species has uncovered substantial cryptic diversity, including the revision of 175 morphospecies of avian malaria parasites to an estimated 10,000 using mitochondrial (mt) DNA markers [14]. However, the division of nominal parasite species into cryptic species complexes has significance beyond the contribution to our knowledge of parasite biodiversity. Cryptic parasite species may inform our understanding of host-parasite coevolution and cospeciation [15], have different host-invasion pathways [16] and exhibit different degrees of host specificity [9, 17]. Where parasites are vectors of viruses or bacteria, resolving the host range of cryptic species can inform epidemiology [9].

Host specificity is a measure of the frequency with which a parasite species associates with a single host species [18]. Parasites limited to only one host species are host specialists, and less discriminate parasites found in association with multiple host species are host generalists. However, morphological conservatism looms as an alternative explanation for some host-generalist parasites identified solely by morphological attributes [9]. The value of using molecular genetic techniques to delimit levels of host specificity is increasingly recognized [19]. Genetic markers have shown several supposedly generalist parasite “species” to be complexes of cryptic, host-specific species [9, 20]. Accurately determining host specificity of parasite species is of wildlife conservation and human health concerns because generalist parasites may be more capable of vectoring pathogens to novel hosts [21]. Generalist parasites are also more competent invaders of new environments than specialists [21], a trait that warrants attention as anthropogenic change increasingly brings invasive parasites into contact with novel habitats and hosts [22].

Bat flies (Diptera: Streblidae and Nycteribiidae) are obligate, blood-feeding ectoparasites of bats. Species of Nycteribiidae are uniformly spider-like in appearance and wingless, restricting their ability to disperse independently of their hosts [23]. Bat flies reproduce via viviparous pupiparity, and gravid females generally leave the host only to adhere a single pupa to the roost substrate [24]. Bats are known reservoirs for an exceptionally diverse array of pathogens, including the ancestor of all mammalian malarias [25], as well as many zoonotic viruses relevant to human health [26–28]. As obligate parasites feeding exclusively on bat blood, bat flies warrant increased attention as potential vectors of these pathogens [29]. Bat flies have been identified as vectors of protozoan parasites [30] and bacterial pathogens in genus *Bartonella* [31, 32], and have recently been found to harbor bat-associated viruses related to medically impactful zoonoses [33–35].

Bat flies were historically understood to possess relatively low host specificity due to frequent host-switching opportunities, facilitated by polyspecific roosting behavior in bats [36]. They also spend a significant portion of their life-cycle off-host due to pupation on roost substrate [37]. However, most bat fly species are strictly monoxenous, or found in reliable association with only one bat species [29, 38]. In many parasites, patterns of host specificity are governed by host and parasite ecological characteristics facilitating reliable host-parasite encounters and eventual evolutionary associations. However, host specificity in bat flies seems to be unaligned with their host-independent dispersal ability (e.g. vagility) or other ecological associations that could provide host-switching opportunities [37, 39]. High host specificity in bat flies may therefore be maintained by host immunocompatibility or the decreased probability of flies encountering suitable mates on non-primary hosts (“reproductive filter” [37]). Bat flies associated with multiple host species are often stenoxenous or oligoxenous, infesting only closely related or congeneric bats [37]. Previous studies examining genetic variation in oligoxenous bat flies using mitochondrial genetic markers have uncovered little geographic or host-structured population genetic structure [40, 41].

*Penicillidia fulvida* (Diptera: Nycteribiidae) is an African bat fly species that exhibits unusually low host specificity [42] and is thus apparently polyxenous. Collections summarized by Theodor in 1967 associated *P. fulvida* with 14 host species, and noted a wide range of host associations relative to other bat fly species [42]. *Penicillidia fulvida* specimens collected in Kenya and currently stored in the Field Museum of Natural History Collection of Hippoboscoid Diptera (Field Museum of Natural History, Chicago, IL, USA) have been recovered from 14 putative host species spanning seven genera and six families of bat. In contrast to the general pattern of monoxenous or oligoxenous associations of bat flies with host bats [37], *P. fulvida* is known to parasitize bat families that diverged over 60 million years ago [43]. The rarity of polyxenous host associations among bat flies, in concert with the increasingly recognized prevalence and ecological importance of cryptic parasite species, suggests that *P. fulvida* may represent a complex of cryptic, and possibly more host-specific, bat fly species. Alternatively, *P. fulvida* may truly represent a single generalist species capable of colonizing distantly related hosts. The questions addressed in this study are: (i) do patterns of genetic differentiation exist within *P. fulvida*, potentially indicative of cryptic speciation? and (ii) is there genetic differentiation of *P. fulvida* parasitizing sympatric hosts, which might indicate cryptic host specificity?

## Methods

### Sampling

All *P. fulvida* specimens used in this study were collected during field expeditions of the “Bats of Kenya” project between 2006 and 2015. This survey was designed to inventory Kenya’s diverse habitats and rich faunas, and much remains to be learned about the limits and interrelationships of bat species. The project assayed genetic variation in 21 bat genera belonging to 10 families, identifying putative species using both mitochondrial and nuclear markers (e.g. [44–47]). To avoid adding to current taxonomic confusion, a conservative approach was purposefully taken regarding the assignment of names to clades in the analyses. Where a clade’s taxonomic identity was ambiguous or unknown, it was simply labeled as a numbered clade, and this convention is reflected in identifying *P. fulvida* hosts. Integrative taxonomic diagnoses of the various clades are needed to determine which (if any) existing names may apply to them.

**Fig. 1:**
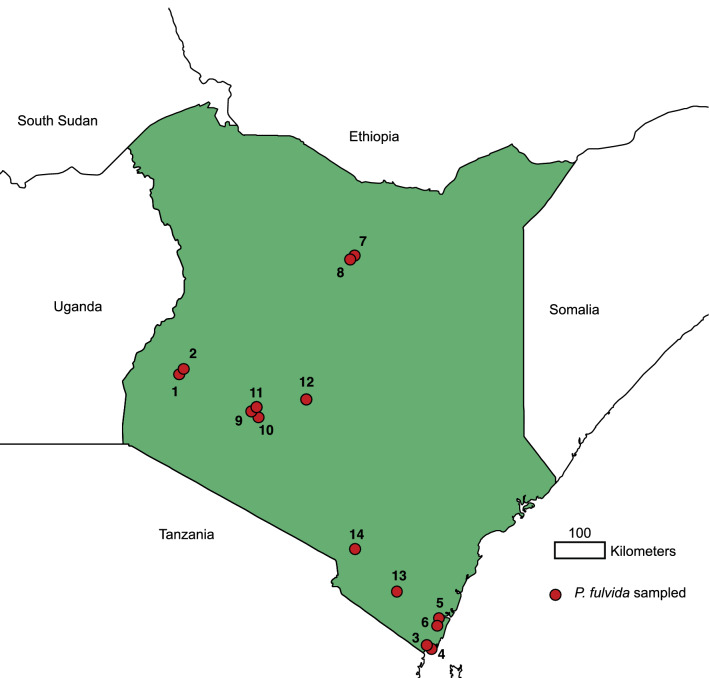
Map of Kenya featuring the 14 sampling localities yielding *Penicillidia fulvida*. A gazetteer of sampling localities is provided in Table 1

**Fig. 2:**
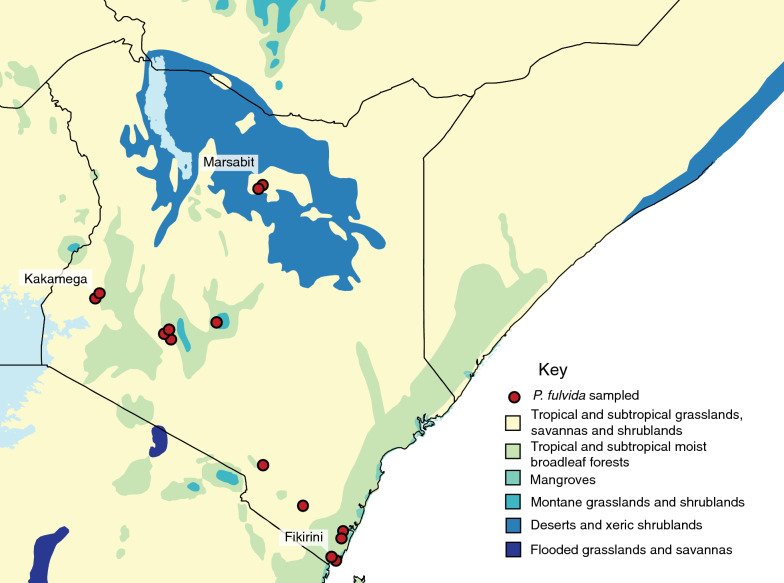
Map of Kenya featuring 14 sampling localities yielding *P. fulvida* and biomes as delineated by Olson et al. [82]

*Penicillidia fulvida* were recovered from 14 localities, comprising mostly tropical broadleaf forests and savannas in the southern and western regions of Kenya (Figs. 1, 2). A total of 65 *P. fulvida* specimens were collected (Table 1); all are referenced in this study to describe host associations (Table 2), and 59 were sequenced. During collection, bats were captured in mist nets or harp traps during foraging/commuting or in hand nets at roosting sites, and all were subsequently stored individually in clean cloth bags to minimize the risk of parasite disturbance transfers. Bats were either safely released or retained as species vouchers after processing. Vouchered bats were euthanized with halothane following guidelines by the American Society of Mammalogists [48], under the approval of the Field Museum’s Institutional Animal Care and Use Committee (2012-003). Voucher specimens were fumigated with ethyl ether to facilitate collection of their ectoparasites, but *P. fulvida* were also reliably detected and extracted from live bats because of their large size. Once extracted from the host, bat flies were immediately stored in 95% ethanol and later identified under a light microscope using species keys and descriptions from Theodor [42] as well as reference specimens from the Field Museum collection. All fly specimens are currently housed in the Field Museum of Natural History Collection of Hippoboscoid Diptera (currently on long-term loan to C. W. Dick at Western Kentucky University).

**Table 1:** Summary of Kenyan *Penicillidia fulvida* (*n* = 65) collected, including host identity, collection locality and coordinates, specimens used for sequencing and cytochrome* c* oxidase subunit 1 haplotype groups

Bat fly ID	Host family	Host species	Locality description	Locality	Latitude	Longitude	*COI*	*COI* haplotype	*28S*
FMNH 215683	Miniopteridae	*Miniopterus* clade 10	Kakamega Forest Station, Lirhanda Hill Cave	1	0.218	34.897	Yes	7	Yes
FMNH 215677	Miniopteridae	*Miniopterus* clade 10	Kakamega Forest Station, Lirhanda Hill Cave	1	0.218	34.897	Yes	8	Yes
FMNH 215707	Miniopteridae	*Miniopterus* clade 10	Kakamega Forest, Mahiakalo Cave	2	0.244	34.907	Yes	1	Yes
FMNH 215683	Miniopteridae	*Miniopterus* clade 10	Kakamega Forest, Mahiakalo Cave	2	0.244	34.907	Yes	2	Yes
FMNH 215719	Miniopteridae	*Miniopterus* clade 10	Kakamega Forest, Mahiakalo Cave	2	0.244	34.907	Yes	6	Yes
FMNH 215706	Miniopteridae	*Miniopterus* clade 10	Kakamega Forest, Mahiakalo Cave	2	0.244	34.907	Yes	2	Yes
NMK 184895	Miniopteridae	*Miniopterus* clade 10	Kakamega Forest, Mahiakalo Cave	2	0.248	34.907	No	-	Yes
FMNH 220168	Rhinolophidae	*Rhinolophus* *fumigatus* 8	Fikirini, Pare Cave	3	− 4.59	39.331	Yes	1	Yes
FMNH 220543	Miniopteridae	*Miniopterus* clade 2	Fikirini, Pare Cave	3	− 4.59	39.331	Yes	6	Yes
FMNH 220544	Miniopteridae	*Miniopterus* clade 2	Fikirini, Pare Cave	3	− 4.59	39.331	Yes	6	Yes
FMNH 220519	Miniopteridae	*Miniopterus* clade 2	Fikirini, Three Sisters, Kisimani Cave	4	− 4.615	39.353	Yes	6	Yes
FMNH 220524	Miniopteridae	*Miniopterus* clade 2	Fikirini, Three Sisters, Kisimani Cave	4	− 4.615	39.353	Yes	6	Yes
FMNH 220254	Hipposideridae	*Triaenops* *afer*	Fikirini, Three Sisters, Mbenyenye Cave	4	− 4.61	39.354	Yes	6	Yes
FMNH 220517a	Miniopteridae	*Miniopterus* clade 2	Fikirini, Three Sisters, Mbenyenye Cave	4	− 4.61	39.354	Yes	6	Yes
FMNH 220517b	Miniopteridae	*Miniopterus* clade 2	Fikirini, Three Sisters, Mbenyenye Cave	4	− 4.61	39.354	Yes	6	Yes

Bat fly ID	Host family	Host species	Locality description	Locality	Latitude	Longitude	*COI*	*COI* haplotype	*28S*
FMNH 220371	Emballonuridae	*Taphozous* *hildegardeae*	Mwaluganje Community Elephant Sanctuary, Ngomeni Cave	5	− 4.082	39.483	Yes	3	Yes
FMNH 220560	Miniopteridae	*Miniopterus* clade 3	Mwaluganje Community Elephant Sanctuary, Ngomeni Cave	5	− 4.082	39.483	Yes	6	Yes
FMNH 220464	Nycteridae	*Nycteris* *thebaica* 4	Shimba Hills National Reserve, Sable Bandas	6	− 4.215	39.451	Yes	9	No
NMK 184220	Rhinolophidae	*Rhinolophus* *cf.* *landeri*	Marsabit National Park and Reserve, southeast	7	2.32	37.994	Yes	10	Yes
NMK 184392	Rhinolophidae	*Rhinolophus* *fumigatus* 3	Marsabit National Park and Reserve, southeast	7	2.309	38	Yes	10	Yes
NMK 184197	Rhinolophidae	*Rhinolophus* *fumigatus* 3	Marsabit National Park and Reserve, southeast	7	2.32	37.994	Yes	10	Yes
NMK 184246	Rhinolophidae	*Rhinolophus* *fumigatus* 3	Marsabit National Park and Reserve, southwest	8	2.283	37.954	Yes	10	Yes
NMK 184251	Rhinolophidae	*Rhinolophus* *fumigatus* 2-3	Marsabit National Park and Reserve, southwest	8	2.283	37.954	Yes	10	Yes
NMK 184242a	Rhinolophidae	*Rhinolophus* *fumigatus* 3	Marsabit National Park and Reserve, southwest	8	2.283	37.954	Yes	10	Yes
NMK 184242b	Rhinolophidae	*Rhinolophus* *fumigatus* 3	Marsabit National Park and Reserve, southwest	8	2.283	37.954	Yes	2	Yes
NMK 182293	Miniopteridae	*Miniopterus* clade 1	Gilgil, Kariandusi Mines	9	− 0.451	36.282	Yes	6	Yes
NMK 182292a	Miniopteridae	*Miniopterus* clade 1	Gilgil, Kariandusi Mines	9	− 0.451	36.282	Yes	12	Yes
NMK 182292b	Miniopteridae	*Miniopterus* clade 1	Gilgil, Kariandusi Mines	9	− 0.451	36.282	Yes	1	Yes
NMK 184700	Miniopteridae	*Miniopterus* clade 1 or 4	Gilgil, Kariandusi Mines	9	− 0.451	36.282	Yes	4	Yes
NMK 184688	Miniopteridae	*Miniopterus* clade 1	Gilgil, Kariandusi Mines	9	− 0.451	36.282	Yes	6	Yes
NMK 184669	Rhinolophidae	*Rhinolophus* *cf.* *landeri*	Gilgil, Kariandusi Mines	9	− 0.451	36.282	Yes	6	Yes
NMK 184693	Miniopteridae	*Miniopterus* clad*e* 1 or 4	Gilgil, Kariandusi Mines	9	− 0.451	36.282	Yes	6	Yes
NMK 184697	Miniopteridae	*Miniopterus* clade 1 or 4	Gilgil, Kariandusi Mines	9	− 0.451	36.282	Yes	6	Yes
NMK 184698	Miniopteridae	*Miniopterus* clade 1 or 4	Gilgil, Kariandusi Mines	9	− 0.451	36.282	Yes	6	Yes
NMK 184792	Rhinolophidae	*Rhinolophus* *clivosus* 2	Gilgil, Kariandusi Mines	9	− 0.451	36.282	Yes	6	Yes
NMK 184689a	Miniopteridae	*Miniopterus* clade 1	Gilgil, Kariandusi Mines	9	− 0.451	36.282	Yes	1	Yes
NMK 184689b	Miniopteridae	*Miniopterus* *clade* 1	Gilgil, Kariandusi Mines	9	− 0.451	36.282	Yes	1	Yes
NMK 182288	Rhinolophidae	*Rhinolophus* *clivosus* 2	Gilgil, Kariandusi Mines	9	− 0.451	36.282	Yes	6	Yes
NMK 182315	Miniopteridae	*Miniopterus* clade 1	Gilgil, Kariandusi Mines	9	− 0.451	36.282	Yes	6	Yes
NMK 184757	Miniopteridae	*Miniopterus* *clade* 4 or 7	Gilgil, Pipeline Cave	10	− 0.539	36.294	Yes	5	Yes
NMK 184754	Miniopteridae	*Miniopterus* clade 4 or 7	Gilgil, Pipeline Cave	10	− 0.539	36.294	Yes	6	Yes
NMK 184833	Rhinolophidae	*Rhinolophus* *fumigatus* 4	Gilgil, Pipeline Cave	10	− 0.539	36.294	No	-	No
FMNH 234882	Miniopteridae	*Miniopterus* clade 8	Soysambu Conservancy, Diatomite Cave	11	− 0.430	36.174	Yes	11	Yes
FMNH 225833	Vespertilionidae	*Myotis* *tricolor*	Soysambu Conservancy, Monkey Bridge Campsite	11	− 0.392	36.211	Yes	6	Yes
NMK 184679	Vespertilionidae	*Myotis* *tricolor*	Menengai crater, Mau Mau cave	12	− 0.217	37.137	Yes	6	Yes
NMK 184709	Miniopteridae	*Miniopterus* clade 1 or 4	Menengai crater, Mau Mau cave	12	− 0.217	37.137	Yes	6	Yes
NMK 184717a	Miniopteridae	*Miniopterus* clade 1 or 4	Menengai crater, Mau Mau cave	12	− 0.217	37.137	Yes	6	Yes
NMK 184717b	Miniopteridae	*Miniopterus* clade 1 or 4	Menengai crater, Mau Mau cave	12	− 0.217	37.137	Yes	6	Yes
FMNH 234771	Miniopteridae	*Miniopterus* clade 1 or 4	Menengai crater, Mau Mau cave	12	− 0.217	36.055	Yes	6	Yes
FMNH 225826a	Vespertilionidae	*Myotis* *tricolor*	Menengai crater, Mau Mau cave	12	− 0.252	36.055	Yes	1	Yes
FMNH 225826b	Vespertilionidae	*Myotis* *tricolor*	Menengai crater, Mau Mau cave	12	− 0.252	36.055	Yes	6	Yes
FMNH 225828a	Vespertilionidae	*Myotis* *tricolor*	Menengai crater, Mau Mau cave	12	− 0.252	36.055	No	-	No
FMNH 225828b	Vespertilionidae	*Myotis* *tricolor*	Menengai crater, Mau Mau cave	12	− 0.252	36.055	Yes	1	Yes
FMNH 225829	Vespertilionidae	*Myotis* *tricolor*	Menengai crater, Mau Mau cave	12	− 0.252	36.055	Yes	6	Yes
FMNH 225907	Miniopteridae	*Miniopterus* clade 1 or 4	Menengai crater, Mau Mau cave	12	− 0.252	36.055	Yes	6	Yes
FMNH 225830	Vespertilionidae	*Myotis* *tricolor*	Menengai crater, Mau Mau cave	12	− 0.252	36.055	Yes	1	Yes
FMNH 225825	Vespertilionidae	*Myotis* *tricolor*	Menengai crater, Mau Mau cave	12	− 0.252	36.055	Yes	6	Yes
FMNH 225824	Vespertilionidae	*Myotis* *tricolor*	Menengai crater, Mau Mau cave	12	− 0.252	36.055	Yes	6	Yes
NMK 184705	Miniopteridae	*Miniopterus* clade 1 or 4	Menengai crater, Mau Mau cave	12	− 0.217	37.137	Yes	6	Yes
NMK 184712	Miniopteridae	*Miniopterus* clade 1 or 4	Menengai crater, Mau Mau cave	12	− 0.217	37.137	Yes	6	Yes
FMNH 216012-013a	–	*Pipistrellus* sp. or *Coleura* *afra*	Marungu Cave	13	− 3.61	38.74	Yes	1 or 2	Yes
FMNH 216012-013b	–	*Pipistrellus* sp. or *Coleura* *afra*	Marungu Cave	13	− 3.61	38.74	No	–	No
FMNH 215994	Emballonuridae	*Coleura* *afra*	Marungu Cave	13	− 3.61	38.74	No	–	No
FMNH 216021	Emballonuridae	*Coleura* *afra*	Tsavo West National Park, Shetani Caves	14	− 2.855	38.001	No	–	No
FMNH 216015	Emballonuridae	*Coleura* *afra*	Tsavo West National Park, Shetani Caves	14	− 2.855	38.001	No	–	No
FMNH 216023	Emballonuridae	*Coleura* *afra*	Tsavo West National Park, Shetani Caves	14	− 2.855	38.001	No	–	No

*COI* Cytochrome* c* oxidase subunit 1,* FMNH* Field Museum of Natural History Collection,* NMK* National Museums of Kenya

**Table 2:** Concise summary of 63 *P. fulvida* host associations in Kenya

Host family	No. of *P. fulvida* specimens^a^	Host species	No. of *P. fulvida*	Percentage total associations^b^
Miniopteridae	34	*Miniopterus* clade 1	7	11.1
		*Miniopterus* clade 1 or 4	11	17.5
		*Miniopterus* clade 10	6	9.5
		*Miniopterus* clade 2	6	9.5
		*Miniopterus* clade 3	1	1.6
		*Miniopterus* clade 4 or 7	2	3.2
		*Miniopterus* clade 8	1	1.6
Rhinolophidae	12	*Rhinolophus clivosus* 2	2	3.2
		*Rhinolophus fumigatus* 2–3	1	1.6
		*Rhinolophus fumigatus* 3	5	7.9
		*Rhinolophus fumigatus* 4	1	1.6
		*Rhinolophus fumigatus* 8	1	1.6
		*Rhinolophus* cf. *landeri*	2	3.2
Vespertilionidae	10	*Myotis tricolor*	10	15.9
Emballonuridae	5	*Coleura afra*	4	6.3
		*Taphozous hildegardeae*	1	1.6
Rhinonycteridae	1	*Triaenops afer*	1	1.6
Nycteridae	1	*Nycteris thebaica*	1	1.6

^a^Two specimens with uncertain host associations (BDP4273-4a and b) are excluded

^b^Proportionally, *Miniopterus* “clade 1 or 4” and *Myotis tricolor* hosted the most *P. fulvida* (17.5% and 15.9% respectively)

Two congeneric African bat flies, *Penicillidia pachymela* and *P. leptothrinax*, were also sequenced to better gauge “species-level” divergence in target genes. *Penicillidia pachymela* is posited to be closely related to *P. fulvida* based on morphological characteristics, and is also assigned to the *P. fulvida* group of species [42]. *Penicillidia pachymela* was collected in sympatry with *P. fulvida* but is exceedingly rare across their shared range in Kenya, with only a single specimen collected during this cumulative 9-year sampling effort. *Penicillidia leptothrinax* is a more distantly related and smaller-bodied species endemic to Madagascar.

### DNA extraction, amplification and sequencing

One or two legs were removed from each *P. fulvida* specimen for genetic analysis to allow retention of morphological vouchers. Prior to DNA extraction, each leg was lacerated with sterile forceps to expose the muscle tissue beneath the exoskeleton and transferred to a 1.5-ml microcentrifuge tube. Whole genomic DNA extractions were performed according to the manufacturer’s protocol using the DNeasy Blood and Tissue Kit (Qiagen, Hilden, Germany), with the final elution divided into two steps at volumes of 35 and 65 μl, respectively, to optimize DNA concentration. All extractions were assessed for quality using a NanoDrop 2000 spectrophotometer (Thermo Fisher Scientific, Waltham, MA, USA).

A 658-bp fragment of the cytochrome* c* oxidase subunit I (*COI*) gene was amplified using the invertebrate-specific primer pair LCO1490 (5’-GGTCAACAA ATCATAA-AGATATTGG-3’) and HCO2198 (5’-TAACTTCAGGGT GACCAAAAAATCA-3’) [49]. Each PCR assay was conducted in a total reaction volume of 25 μl containing 12.5 μl GoTaq MasterMix (Promega, Madison, WI, USA), 9 μl of nuclease-free water, 1 μl each of 10 μM forward and reverse primers and 1.5 μl of DNA template. A negative control, with nuclease-free water replacing the template DNA, was included with every set of reactions. Thermal cycling conditions for *COI* consisted of an initial denaturing at 95 °C for 1 min, followed by 35 cycles of 95 °C for 2 min, 50 °C and 72 °C for 1 min, with a final extension at 72 °C for 5 min.

MtDNA has rapid substitution rates, making mitochondrial sequence data favorable for parsing relatively shallow, species-level phylogenetic relationships [50]. Although segments of *COI* have been proposed as “universal barcodes” suitable for species delimitation almost ubiquitously across taxa [50], sole reliance on mitochondrial markers disregards other modes of inheritance [51] and can obscure potential effects of introgression or infection with *Wolbachia*, an arthropod-associated bacterial endosymbiont capable of disrupting patterns of mitochondrial inheritance [52]. For a multilocus approach, we also amplified *28S* ribosomal RNA (rRNA), a nuclear gene commonly used alongside *COI* for species-level analyses in arthropods [53].

The D2 region of the nuclear *28S* gene was amplified using the primers F2 (5-AGAGAGAGTTCAAGAGTACGTG-3’) and 3DR (5’-TAGTTCACCATCTTTCGGGTC-3’) [54]. Reaction components and volumes were identical to those used to amplify *COI*. Thermal cycling conditions for *28S* were consisted of an initial denaturing at 98 °C for 2 min, followed by 35 cycles of denaturing at 94 °C for 3 min, annealing at 51 °C for 30 s, with a first extension at 72 °C for 2 min and then a final extension at 72 °C for 8 min.

PCR products were verified for size and quality via electrophoresis on a 1.5% agarose gel stained with SybrSafe (Invitrogen, Thermo Fisher Scientific, Waltham, MA, USA) and visualized under a blue LED light. Sanger sequencing was performed at the North Carolina State Genomic Sciences Laboratory (Raleigh, NC, USA) using forward and reverse primers for both genes.

### Phylogenetic analyses

Sequences were trimmed and assessed for ambiguous bases by eye, then aligned using the MUSCLE algorithm [55] as implemented in Geneious 6.0 [56]. A hippoboscid fly, *Pseudolynchia canarensis*, was included as an outgroup (sequence data retrieved from GenBank). Haplotype groups were identified using DnaSP 6 [57], and a minimum-spanning haplotype network was constructed with PopART [58].

Substitution models for Bayesian and maximum likelihood analyses were estimated with jModeltest [59]. Optimized substitution models based on Akaike’s information criterion were GTR + G for *COI* and HKY + I for *28S*. Both concatenated and single-gene trees were created, with concatenated trees partitioned by locus. Bayesian analysis was performed in MrBayes 3.2 [60] using the default burn-in of 25% and 10 million Markov chain Monte Carlo generations. Average standard deviation in split frequencies fell below 0.01 at 380,000 then at 650,000 generations and remained under this threshold until the final generation, indicating stationarity was reached. Posterior probabilities for clade support were calculated using the trees remaining after burn-in. A maximum likelihood phylogeny was created using RAxML 8.0 [61]. The starting tree was obtained by searching for the best-scoring maximum likelihood tree in a single program run. Branch support was calculated using a random seed and 1000 rapid bootstrap replicates.

### Analysis of molecular variance

A hierarchical analysis of molecular variance (AMOVA) was used to determine the extent to which genetic variation in *P. fulvida* is allocated among host taxa. Because AMOVA requires groups to be defined a priori, haplotypes were conservatively pooled by host family. Host families with insufficient sample sizes for statistical analysis (Emballonuridae, Nycteridae and Hipposideridae, from which only 1–3 *P. fulvida* specimens were recovered or sequenced) were excluded; therefore, only specimens from Miniopteridae (*n* = 33), Rhinolophidae (*n* = *11*) and Vespertilionidae (*n* = 9) were compared. AMOVA was performed in Arlequin 3.5.2.2 [62] using a locus-by-locus method to accommodate missing data. The significance of pairwise fixation indices (F_st_, an F-statistic measuring variance in allele frequency among populations) was calculated using 1023 permutations, each assigning parasite haplotypes to host families at random to generate a null distribution.

## Results

### Phylogenetic analyses

**Fig. 3:**
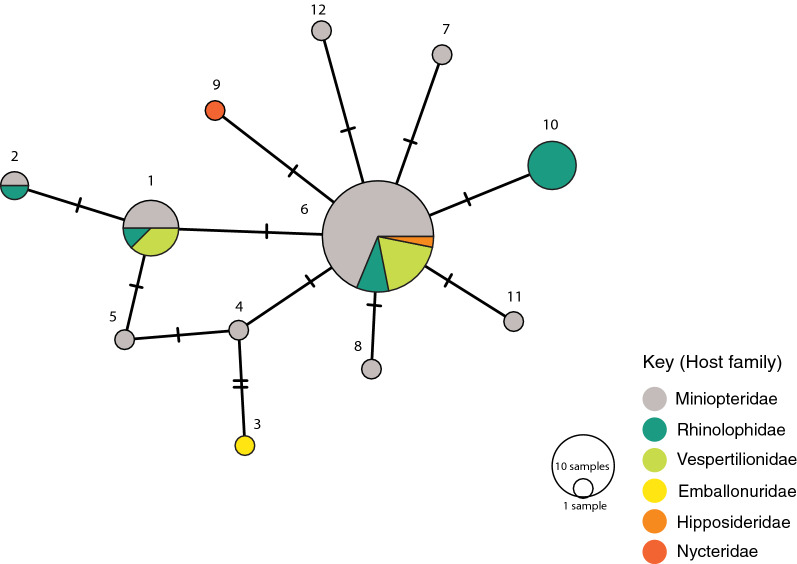
Minimum-spanning haplotype network of 12 unique *P. fulvida* cytochrome* c* oxidase subunit 1 (*COI*) haplotypes, allocated by host family

A total of 12 *COI* haplotypes were present within *P. fulvida*, with 11 variable sites (Fig. 3). One specimen (FMNH216012-013) could not be assigned a *COI* haplotype due to an ambiguous base at a variable site, and was excluded from the haplotype network. Only a single *28S* haplotype was recovered from *P. fulvida*, but *28S* haplotypes were distinct among the three described species within *Penicillidia* (1.8% pairwise uncorrected distance [p-distance] between *P. leptothrinax* and the most common *fulvida* haplotype, 1.6% p-distance between *P. pachymela* and *P. fulvida* and 3.2% p-distance between *P. leptothrinax* and *pachymela*). The concatenation of *28S* with *COI* in phylogenetic analyses yielded poorly resolved relationships among the three putative species and was too conservative to inform population genetic structure within *P. fulvida*. Because *P. pachymela* and *P. leptothrinax* had only single specimens included for comparative purposes and, in addition, resolving relationships among putative species within *Penicillidia* is outside the scope of this study, only *COI*-based trees were included.

**Fig. 4:**
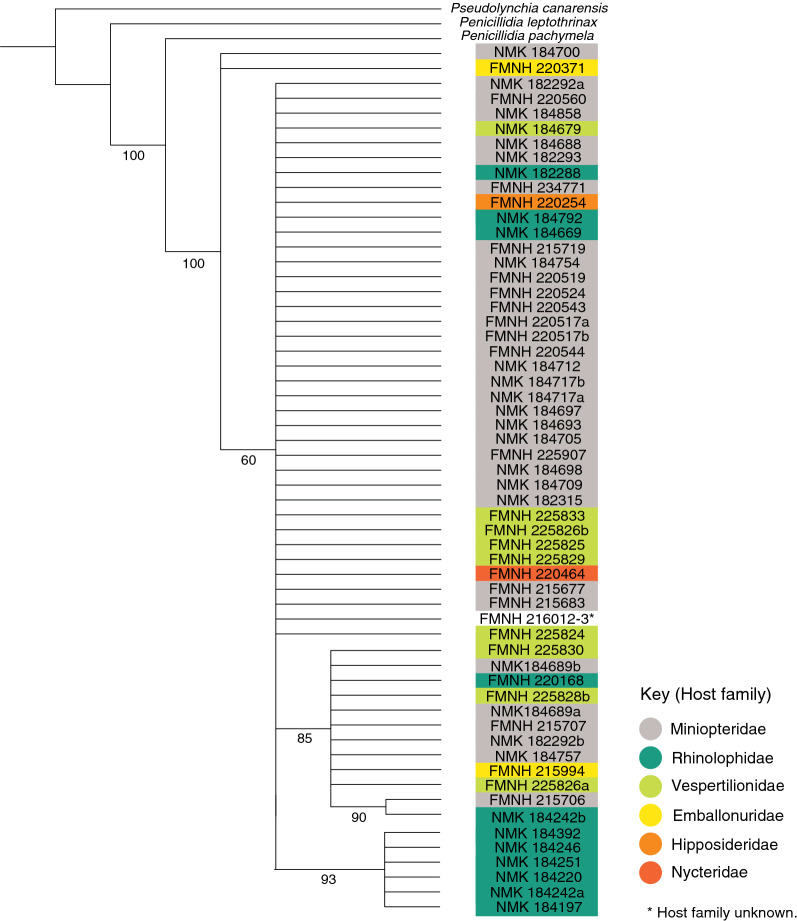
Bayesian phylogenetic tree (*P. fulvida* labeled with voucher identifications (IDs), congeners *Penicillidia pachymela* and *P. leptothrinax* and outgroup *Pseudolynchia canarensis*) constructed using *COI*, a GTR + G substitution model and 10 million generations. Posterior probabilities for clade support are presented on a scale of 0–100 for ease of comparison to maximum likelihood bootstrap values

**Fig. 5:**
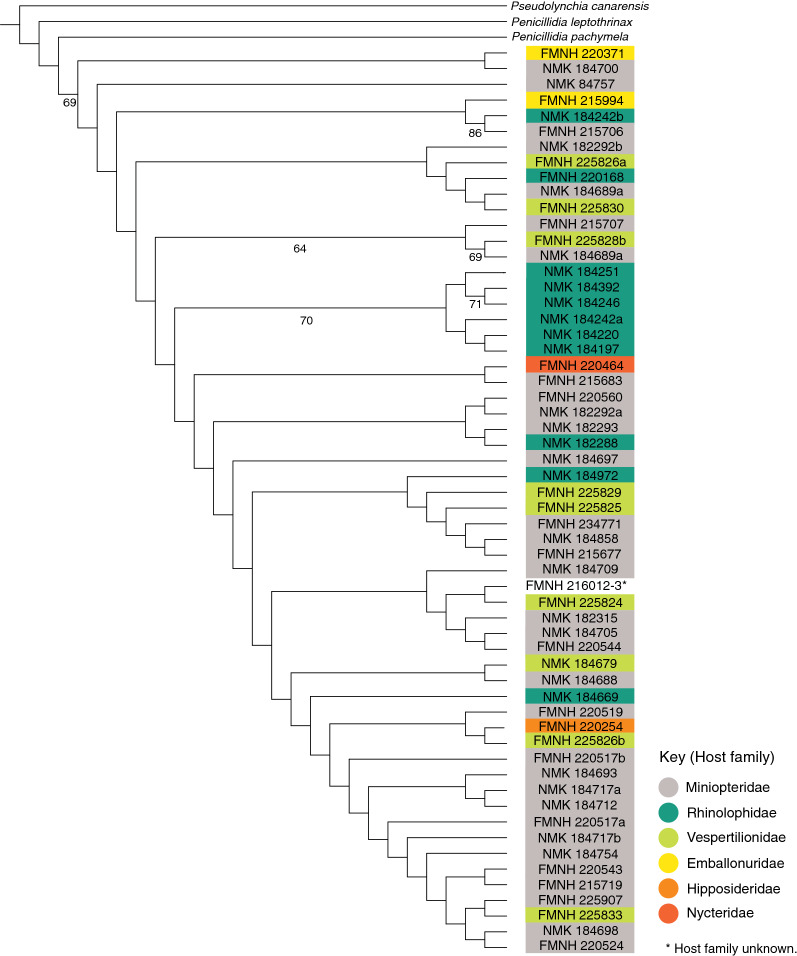
Maximum likelihood tree (*P. fulvida* labeled with voucher IDs, congeners *P. pachymela* and *P. leptothrinax* and outgroup *Pseudolynchia canarensis*) generated using *COI* and a GTR + G substitution model. Branch support was calculated using 1000 rapid bootstrap replicates, and only support values > 60% are displayed

Bayesian and maximum likelihood analyses yielded incongruent topologies within *P. fulvida* (Figs. 4, 5). The clades that are moderately well-supported but recovered using only a single method lack apparent host-based or geographic structure, and therefore do not appear to warrant discussion. One clade was supported by both Bayesian and maximum likelihood analyses, comprising six specimens from the host family Rhinolophidae and from a single, distant locality (Marsabit; Table 1; Figs. 4, 5). Neither analysis recovered outgroups within the in-group of *P. fulvida*.

### Analysis of molecular variance

Hierarchical AMOVA rejected the null hypothesis of haplotype homogeneity among host families (*P* < 0.001; Table 3). The significance of pairwise *F*_st_ values indicated that allele frequencies were unique in *P. fulvida* haplotype groups from rhinolophid bats (Yinpterochiroptera: Rhinolophoidea) when compared to haplotype groups from both miniopterid and vespertilionid bats (Yangochiroptera: Vespertilionoidea) (Table 4). However, this taxonomic association appears artefactual when regarded alongside phylogenetic structure and sampling composition. Genetic structure associated with Rhinolophidae aligns more reliably with sampling locality (Table 1; Fig. 4); 7 of 11 *P. fulvida* recovered from rhinolophid bats were recovered from Marsabit, and 6/6 of the “rhinolophid” clade were from Marsabit bats). Viewed in this light, the results do not suggest host-based genetic differentiation is present within *P. fulvida*.

**Table 3:** Analysis of molecular variance of genetic structure in *P. fulvida* as it corresponds to host family

Source of variation	Sum of squares	Variance	Percent variation	F-statistic	*P*-value
Among host families	5.804	0.08851	19.42387	0.19424	< 0.001
Within host families	37.818	0.36717	80.57613

**Table 4:** Pairwise F_st_ values among host families of *P. fulvida*

	Rhinolophidae	Miniopteridae	Vespertilionidae
Rhinolophidae		0.26579*	0.27479*
Miniopteridae	0.26579*		0.01145
Vespertilionidae	0.27479*	0.01145	

*Significant at* P *< 0.05 in permutation test.

## Discussion

Parasites are susceptible to placement in artificial species groups because of their morphological conservatism relative to their hosts [9]. The advent of molecular genetic techniques has provided a valuable strategy for scrutinizing morphologically indistinguishable but ecologically differentiated parasites, and can result in the division of host-generalist nominal species into more host-specific cryptic species complexes [9, 14, 15, 17]. Using two genetic markers (mitochondrial *COI* and nuclear *28S*), we have uncovered no such evidence of host-mediated genetic structure in the polyxenous bat fly *P. fulvida*. This finding is concordant with other studies evaluating patterns of population genetic structure in non-monoxenous (oligoxenous) nycteribiid bat flies [40, 41], but to our knowledge represents the first such investigation of a truly polyxenous bat fly species. These results suggest inter-host gene flow may be occurring in *P. fulvida,* and support its recognition as a single species under both morphological and phylogenetic species concepts. Unlike most bat flies, it is capable of parasitizing phylogenetically disparate bat species, and in Kenya parasitizes both suborders (Yinpterochiroptera and Yangochiroptera) and six different families of bats.

Although no genetic structure could be reliably attributed to host identity, there is some evidence *P. fulvida* is not uniformly panmictic across Kenya. Both maximum likelihood and Bayesian phylogenetic analyses supported a clade of 6/7 *P. fulvida* from a single remote sampling site in Marsabit Forest (Figs. 3, 4). Isolation by distance alone is insufficient to explain this geographic differentiation, as *P. fulvida* is essentially panmictic (with respect to the localities sampled) across the comparable 700 km distance between Kakamega and Fikirini (Figs. 1, 2). Kenya is composed of diverse biomes, and Marsabit Forest is well-representative of this mosaic: a tropical broadleaf forest sustained by volcanic soil and orographic precipitation that is completely surrounded by swaths of rocky desert (Fig. 2). Further, all *P. fulvida* from Marsabit Forest were collected from horseshoe bats (5/6 from *Rhinolophus fumigatus* [clade 2–3; see [45]], and 1/6 from *Rhinolophus* cf. *landeri*); all have wings with relatively low aspect ratios, marking them as relatively weak fliers [63]. Marsabit Forest’s geographic isolation may stymie gene flow in *P. fulvida* by restricting the dispersal of its bat hosts. However, given the small sample size and the lack of intervening sampling sites that might firmly implicate the surrounding desert as a barrier to gene flow, the origin of this site-related genetic structure remains speculative.

One specimen of *Rhinolophus fumigatus* from Marsabit hosted two distinct haplotypes of *P. fulvida*: the typical Marsabit-associated clade and a haplotype also found on a *Miniopterus* host at Kakamega Forest over 400 km distant. This suggests lack of barriers between Marsabit and other populations, but absent additional data this finding cannot be explored in more detail.

An alternative explanation for the lack of genetic structure in *P. fulvida* is insufficient variety and variability in the genes used. Nuclear *28S* was conservative among the three *Penicillidia* species sequenced and invariant in *P. fulvida*. Consequently, only mitochondrial *COI* was used to assess patterns of intraspecific, inter-host variation in *P. fulvida*. Drawing conclusions from one mitochondrial gene can be precarious, as potential mito-nuclear discordance and the small proportion of the overall genome represented in single-gene phylogenies can cause incongruence [64]. Further, reliance on mtDNA is unfavorable in some insect taxa, including bat flies, due to possible infection with bacterial *Wolbachia* [65]. *Wolbachia* is maternally transmitted and can cause disruptions in mitochondrial inheritance, resulting in the overrepresentation of certain haplotypes in a population or the absence of polymorphism altogether [52, 66]. The use of more variable nuclear genetic markers in future studies, alongside increased research efforts investigating the ability for *Wolbachia* to influence inheritance in nycteribiid bat flies, could help clarify the possibility of *Wolbachia* infection obscuring genetic structure in *P. fulvida*.

Understanding host specificity and the processes responsible for its evolution and maintenance across parasite communities is crucial to understanding the evolutionary context of host-parasite associations and the role of parasites in disease transmission. High host specificity in parasites has historically been regarded as the default trend in parasite evolution; specialization is also regarded as an evolutionary “dead end” because morphological specialization required for a highly specific parasite to efficiently exploit its host makes a specialist lineage’s “return” to a generalist strategy improbable [67]. However, the evolution of ecological resource specialization is not characterized by fixed directionality: different degrees of host specificity can arise multiple times in the evolutionary history of a parasite, with generalist species sometimes emerging from specialist lineages [67–69]. Extensive biodiversity surveys have found bat flies to possess considerably high host specificity as a group, and some instances of low specificity in bat flies may be attributable to poorly understood species boundaries [29]. Using molecular techniques, this study demonstrates that, although apparently rare, polyxenous bat fly species do exist. If *P. fulvida* evolved from specialist ancestors, the ecological precedent for a decrease in specificity across evolutionary time may be resource breadth-associated performance trade-offs [70, 71] or interspecific competition [69].

Host ecology serves as an important evolutionary driver of patterns of host specificity in parasite communities. Multiple bat species often aggregate closely in a single roost, a behavior which may confer anti-predator benefits or simply reflect limited availability of suitable roosts [72, 73]. Mixed-species groups present host-switching opportunities for parasites that depend on their hosts for dispersal; as a result, polyspecific bat roosts were a historical precedent for proposing universally low host specificity in bat flies [36, 74]. *Penicillidia fulvida* was collected from multiple host species in eight of 14 roosts and from multiple host families in seven of 14 roosts in Kenya, indicating polyspecific roosting behavior may be a proponent of *P. fulvida*’s broad host range and high genetic structure within hosts. Further, although it is now recognized that specialist bat flies can maintain their high specificity despite host-switching opportunities [37], bat assemblages in shared roosts may influence host preference in generalist bat flies [75]. Accordingly, there is some indication that parasitism by *P. fulvida* is based on host availability. *Penicillidia fulvida* occasionally parasitizes the African sheath-tailed bat *Coleura afra* (4/63 total host associations in the Bats of Kenya survey; Table 2), but associations with *C. afra* were only recorded in the absence of potential miniopterid and rhinolophid hosts (Table 1). *Coleura afra* was present in three shared roosts containing *Miniopterus* hosts of *P. fulvida*, but *P. fulvida* was never recovered from *C. afra* when these alternative hosts were available. This pattern suggests that although host selection in *P. fulvida* is unconstrained by phylogenetic distance, *P. fulvida* may still demonstrate tiers of host preference, which could function to increase fitness by mitigating local competition or selecting optimally nutritious or compatible host blood [29].

Vector ecology is a valuable determinant of pathogen spread and potential zoonotic spillover [76]. Low host specificity in parasites may promote exposure to a wider range of infectious agents and facilitate disease transmission to new hosts and geographic areas [22]. Bats are reservoirs for a broad spectrum of pathogens, a product of immunological or ecological predisposal [77] or a reflection of their high species diversity [28]. Nycteribiid bat flies, including *P. fulvida,* can serve as vectors facilitating the transmission of several bat pathogens: hemosporidian parasites in genus *Polychromophilus* [25, 30] and bacterial bartonellae [31, 32]. Moreover, research linking nycteribiid bat flies to impactful bat-associated viral zoonoses is expanding [33, 35, 78]. Within the scope of this study, Kenyan hosts of *P. fulvida* are known reservoirs of paramyxoviruses (*Miniopterus* spp. [79]), SARS coronaviruses (*Rhinolophus fumigatus* and *R. landeri* [80]) and lyssaviruses (*Miniopterus* spp. [81]).

Because this study failed to detect significant genetic differentiation among *P. fulvida* parasitizing bats across three superfamilies in both suborders, the results clearly demonstrate that *P. fulvida* is a rare polyxenous species of bat fly. However, it is important to acknowledge this study is not comprehensive with respect to *P. fulvida*’s geographic and host range. *Penicillidia fulvida* has a broad range outside Kenya, comprising much of sub-Saharan Africa [42] and possibly Madagascar (C.W. Dick, unpublished data). Additionally, *P. fulvida* was collected from three host families in Kenya with relatively uninformative sample sizes (Emballonuridae, Rhinonycteridae and Nycteridae) and has previously been recorded from Pteropodidae, although this association is tenuously based on a single record (*Eidolon helvum* in South Africa; [42]). Based on associations recorded in this study and past surveys, it seems probable that bats from Miniopteridae, Rhinolophidae and Vespertilionidae represent *P. fulvida*’s primary host range, whereas other, infrequently observed hosts are non-primary associations indicative of natural host-switching events. Increased, targeted sampling efforts may be useful for further parsing host associations and for testing hypotheses associated with geographic differentiation in *P. fulvida*.

## Conclusions

Host specificity is one of the most basic ecological characteristics of parasites and likely subject to strong selection. Accurate measurement of host specificity is vital to understanding parasite biodiversity, vulnerability to population decline and the role of parasites in disease transmission. Moreover, accurately estimating host specificity relies on acknowledging the presence of cryptic diversity and using integrative approaches to delimiting parasite species. This study provides molecular genetic evidence that the nycteribiid bat fly *P. fulvida* does not exhibit cryptic host specificity, and instead represents a single species with a wide range of phylogenetically distant bat hosts.

## Data Availability

All sequence data are available in the GenBank repository (*COI *accession numbers ON704663-ON70421; *28S* accession numbers ON693298-ON693355).
